# Image Fusion and Stylization Processing Based on Multiscale Transformation and Convolutional Neural Network

**DOI:** 10.1155/2022/1181189

**Published:** 2022-04-28

**Authors:** Qingzeng Xu

**Affiliations:** College of Artificial Intelligence, Tianjin University of Science & Technology, Tianjin 300000, China

## Abstract

With the continuous development of imaging sensors, images contain more and more information, the images presented by different types of sensors are different, and the images obtained by the same type of sensors under different parameters or conditions are also different. Multisource image fusion technology combines images acquired by different types of sensors or the same type of sensors with different parameter settings, which makes the image information more complete, compensates for the limitations of images of the same type, and also allows you to save information about the characteristics of the original image. Multimodal image mosaic and multifocal image mosaic have been studied in detail in two directions. On the one hand, a method based on frequency domain transformation is used for multiscale image decomposition. On the other hand, image extraction with neural network-based methods is proposed. The technology of convolutional neural networks (CNNs) allows to extract richer texture features. However, when using this method for fusion, it is difficult to obtain an accurate decision map, and there are artifacts in the fusion boundary. Based on this, a multifocal fusion method based on a two-stage CNN is proposed. Train the advanced intensive network to classify input image blocks as focus, and then use the appropriate merge rules to get the ideal decision tree. In addition, several versions of the fuzzy learning set have been developed to improve network performance. Experimental results show that the frames of the first stage proposed by the algorithm make it possible to obtain an accurate decision scheme and that the frames of the second stage make it possible to eliminate the pseudo-shadow of the integration boundary.

## 1. Introduction

**Figure 1:**
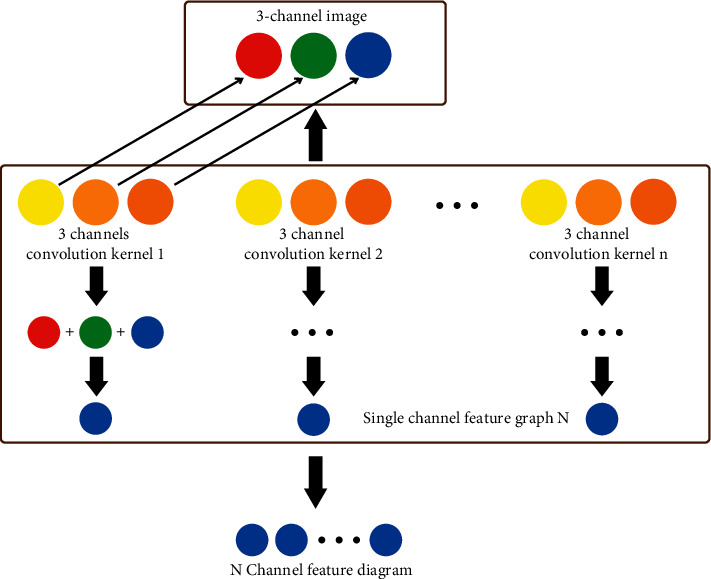
Image fusion processing based on convolutional neural network.

Vision is one of the important ways for humans to perceive the world, understand the world, and explore the world. However, being a reliable carrier of environmental information, the image is subject to such objective realistic factors as the size, color, shape, spatial position of the object-the object, as well as the subjective limitations of the visual system of the human eye, which do not allow obtaining directly and accurately relevant information about the object-the object only through visual observation of the visual system of the human eye, and therefore people tend to use external devices to understand and process some information about the target [1]. In the era of rapid development of information technology, the development of computer vision technology is also very fast. As an important branch of computer vision technology, image fusion technology has made great achievements in just a few decades with the continuous exploration of researchers all over the world. Image fusion is not just an overlay of multiple images, it creates new images with more valuable information [2]. This technology was developed to overcome the limitations of traditional optical lens aperture and other imaging equipment and to take advantage of the complementarity and redundancy of information carried by the multisource images themselves. Special image processing mechanisms combine complementary characteristic components of multisource images, ultimately enabling a more comprehensive and precise representation of all relevant content in the same scene. Image technology is, therefore, a very effective means of image processing and has been successfully applied in infrared detection, remote sensing search, medical imaging, and digital products. In image processor technology, we divide the source image into multiple focused images, medical image groups, telemetry resolutions, and infrared and visible light effects. Multifocus images have long been research centers in the field of image connection [3]. When there are multiple targets in the same scene, the conventional imaging device is limited by their depth of field, single sighting can only make the targets in the focus area into sharp images and blurry images elsewhere, and this special imaging mechanism becomes an obstacle for researchers when observation and analysis of scene information. The emergence of multifocus image fusion technology effectively solves the problem of inconvenient observation caused by image groups with different focal areas; this technology can automatically identify the focus area of each image; the extracted focal area information is fused into an image in which all target objects are clear, detailed information is more abundant, and scene description is more accurate. Multifocus image fusion technology is widely used in military, medical, and imaging equipment development. At the same time, this technology lays a good foundation for subsequent image processing technologies, such as image extraction, target recognition, image classification, and so on, so learning how to improve the algorithm for recognizing the borders of the focus area of images with nonfocus areas and effectively extracting information about the focus area of images are of great importance for the development of multifocus image fusion technology [4]. This paper mainly studies the image fusion processing technology based on convolutional neural network (Figure 1).

## 2. Literature Review

Wang Q. et al. said that with the progress of economy and society, the rapid development of sensor technology, and the increasing number of sensors in application scenarios, the amount of available information is increasing and the forms are more diverse. In order to meet this challenge, information fusion technology came into being [5]. Liang et al. stated that the so-called information fusion refers to the multilevel, multifaceted, and multilevel processing and synthesis of multisource information obtained from multiple sensors in order to obtain richer, more accurate, and more reliable useful information. Image fusion is an important research direction in the field of information fusion, involving sensor technology, artificial intelligence, signal processing, and image processing; it is a comprehensive emerging technology [6]. Singh et al. believed that the so-called image fusion is to use images obtained from different sensors, or images obtained by the same sensor in different imaging methods or at different imaging times, according to certain rules and merge into a new image [7]. The fusion image contains more information than the source image, and the details are richer; the outline is also clearer and can describe the target more accurately, which is convenient for later human observation and computer processing. Zhen et al. proposed that in traditional applications, professional human information observers do this work. Information observers need to observe images of multiple information sources at the same time and use their own visual system and brain to obtain information and make judgments. In this case, the labor load of the observer is large, which is difficult to achieve even after professional training [8]. Azer S. A. et al. stated that multisource image fusion is a new technology to solve this problem. This technology can combine information provided by different types of sensors or different information provided by a single sensor, eliminate redundant information that may exist between information, improve system reliability and serviceability, providing a more accurate, reliable, and comprehensive description of the target or scene for further observation or editing. The combination of CT and MRI images, for example, enables the precise diagnosis of diseases. In Reference [9], Tazeen T. et al. believed that at present, image fusion is not only widely used in military fields such as tracking and recognition, target detection, and situational awareness; it has also been promoted in civil fields such as human visual aids, intelligent transportation, industrial processes, medical imaging and diagnosis, and intelligent manufacturing [10]. Savant et al. stated that in the past 20 years, for image fusion technology at different levels, a lot of in-depth and detailed theoretical and applied research has been carried out. However, so far, the recognized theoretical system and application methods have not yet been formed, and many theoretical and technical problems need to be solved urgently [11]. Bodapati J. D. et al. believed that the research on image fusion technology in China started late and is still backward compared with international research. In terms of basic theory and engineering technology, in-depth research is needed. Image fusion technology refers to combining multiple different images obtained from different sensors or a single sensor multiple times; according to certain rules, it is fused into a new image [12]. Link et al. argued that the adjusted image contains more redundancy and complementary information from the source image, more detailed information, clearer contours, and more accurate target descriptions. Multifocus imaging is one of the research points [13]. Pavan K. et al. stated that according to the principle of convex lens imaging, when the distances between multiple targets and the lens are different, photographic equipment cannot focus these objects on the same focal plane at the same time; that is, they cannot be clearly presented in one image at the same time. Therefore, in order to obtain a clear image of each target object, it is necessary to focus and shoot each object separately [14]. Through the multifocus image fusion technology, the reliability of target detection and recognition and the utilization rate of image information can be effectively improved; therefore, the application of this technology in target recognition, machine vision, digital cameras, and other fields is increasingly widespread.

## 3. Methods

### 3.1. Multisource Image Fusion Technology and Evaluation Indicators

#### 3.1.1. The Concept of Multisource Image Fusion

Image fusion is a way of combining information from multiple images of the same scene, taken from different sensors, in different places or at different times. Fusion images store all the additional and redundant information of the input image, which plays a significant role in the tasks of visual perception and human image processing. Image fusion is a technology for extracting important texture elements from multisource images and integrating them [15]. The formula for the mathematical representation of the fused image is as follows:

(1) $$\begin{array}{c} I_{F}=\phi \left(I_{1},I_{2},I_{3},...,I_{N}\right)=\alpha_{1}I_{1}+\alpha_{2}I_{2}+......+\alpha_{N}I_{N}. \end{array}$$

**Figure 2:**
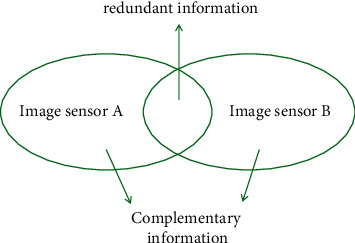
Example of multisource image fusion.

Among them, *I*_1_, *I*_2_, *I*_3_, ..., *I*_*N*_ is the image to be fused, *φ* is the fusion rule, and *a* is a constant and satisfies ∑_*n*=1_^*N*^*α*_*n*_=1.  Figure 2 shows a diagram of the image fusion method. For complete integration of information, the results of integration must meet the following requirements: (1) fusion images must retain additional and important information in the input image; (2) no additional information can be generated during the fusion process; these include manually set information and information obtained through image processing techniques; and (3) registration errors and generated noise must be avoided [16].

#### 3.1.2. An Overview of Traditional Multisource Image Fusion Techniques

The traditional multisource image fusion method allows manual extraction of the characteristics of multimodal images, but with certain limitations. Among them, traditional methods mainly include regional approach, transform field based method, and sparse representation method [17].

**Figure 3:**
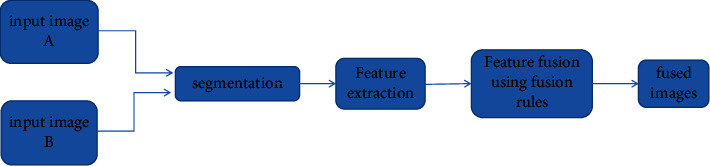
The basic framework of the regional fusion scheme.

1. *Region-Based Multisource Image Fusion Method*. Region-based image integration is done in three different ways. (1) Standard segmentation-based methods first divide the source image into different regions. Then, images are generated using region feature techniques. (2) Statistical and estimation-based methods first use a statistical image-based region segmentation algorithm. Then, the source image is divided into regions. A joint area map is created by parsing the map of each source image, producing a final fusion image. (3) A method based on detection of focal areas and significant diagrams separates important foreground objects from the background and thus forms areas that are perceived as coherent [18]. The basic frames based on the regional integration program are shown in Figure 3. The zone-based image fusion algorithm requires reading two or more input images and then using different partitioning algorithms to separate the images into different regions. Use the appropriate element extraction technology to extract various elements from each area, such as edges, textures, outlines, and more. Finally, use the appropriate merge rules to get the latest image.
2. *Multisource Image Fusion Method Based on Transformation Domain.* Three stages are required to adopt a transform-domain based fusion strategy. First, the image is decomposed using the base wavelet function. Then, the resolved coefficients are consolidated via a prepackaged combination scenario that includes active horizontal surveying, convergence rules, and conformance checking. Finally, using the inverse transform of the multiscale transform, perform a special operation on the merge factor and get the image of the result [19]. Most methods for combining multisourced images based on transform domains include (1) merging images with multiple sources based on pyramid transformation; (2) merging images with multiple sources based on wavelet transform; and (3) image fusion with multiple sources based on the MGA method.

#### 3.1.3. Image Fusion with Multiple Sources Based on Laplacian Pyramid

Laplace Pyramid (LP) was proposed by Burt et al.; the source image can be broken down into subimages with different dimensions and spatial resolutions. The Ralph Pyramid was developed from the Gold Medal, and the Gaussian Pyramid (GP) uses down sampling technology; the image is decomposed at multiple scales; the postdecomposition images are obtained by the following secondary sampling of the images of the previous layer after low-pass filtering [20]. Assuming that the source image *G*_0_ is the 0th layer of the Gaussian pyramid, the image *G*_*l*_ of the *l*-th layer of the Gaussian pyramid is expressed as follows:

(2) $$\begin{array}{c} G_{l}=\sum_{m=-2}^{2}\sum_{n=-2}^{2}\omega \left(m,n\right)G_{l-1}\left(2i+m,2k+n\right)\;0<l\leq N,\leq i<C_{l},0\leq j<R_{l}. \end{array}$$

Among them, *N* is the total number of layers of GP, *C*_*l*_ is the number of columns in layer *l* of GP, *R*_*l*_ is the number of rows in layer *l* of GP, *G*_*l*−1_ is the image of layer *l* − 1, and *ω*(*m*, *n*) is represented as a window function where *ω*(*m*, *n*)=*h*(*m*)*∗h*(*n*), *h* is the Gaussian density distribution function, and the formula of the window function is as follows:

(3) $$\begin{array}{c} \omega =\frac{1}{256}\left[\begin{array}{ccccc} 1 & 4 & 6 & 4 & 1\\ 4 & 16 & 24 & 16 & 4\\ 6 & 24 & 36 & 24 & 6\\ 4 & 16 & 24 & 16 & 4\\ 1 & 4 & 6 & 4 & 1 \end{array}\right]. \end{array}$$

Then, *G*_*l*_ is interpolated and expanded to obtain an enlarged *G*_*l*_^*∗*^, in order to keep the size of *G*_*l*_^*∗*^ consistent with the size of *G*_*l*−1_; the specific formula is as follows:

(4) $$\begin{array}{c} G_{l}^{*}\left(i,j\right)=4\sum_{m=-2}^{2}\sum_{n=-2}^{2}\omega \left(m,n\right)G_{l}\left(\frac{i+m}{2},\frac{j+n}{2}\right). \end{array}$$

Among them, 0 < *l* ≤ *N*, 0 ≤ *i* < *C*_*l*_, 0 ≤ *j* < *R*_*l*_, in the formula as follows:

(5) $$\begin{array}{c} G_{l}^{*}\left(\frac{i+m}{2},\frac{j+n}{2}\right)=\left\{\begin{array}{cc} G_{l}\left(\frac{i+m}{2},\frac{j+n}{2}\right), & w\text{hen }i+\frac{m}{2},j+\frac{n}{2}\text{are integers}\\ 0, & other \end{array}\right. \end{array}$$

Make

(6) $$\begin{array}{c} \left\{\begin{array}{cc} LP_{l}=G_{l}-G_{l+1}^{*}, & 0\leq l<N\\ LP_{N}=G_{N}, & L=N \end{array}\right., \end{array}$$

where *N* is the total number of layers of the *LP* and *LP*_*l*_ is the *l*-th layer image decomposed by the *LP*. Pyramid is formed by *LP*_0_, *LP*_1_, ..., *LP*_*N*_; any layer image of GP is the changed value of the base layer and its previous layer image after expansion [21]. The specific reconstruction process is as follows:

(7) $$\begin{array}{c} \left\{\begin{array}{cc} G_{N}=LP_{N}, & L=N\\ G_{l}=LP_{l}+G_{l+1}^{*}, & 0\leq l<N \end{array}.\right. \end{array}$$

#### 3.1.4. Merge Images with Multiple Sources Based on Wavelet Transform

Image feature is extremely important in many imaging applications; it can be arbitrary in size and can dominate at any particular scale. Therefore, an analysis with multiple resolutions, e.g., a small wave transmission is a very useful mathematical tool for image analysis and computer visualization. A wavelet is a function generated by dilution and transformation from a single prototype function Ψ. The specific form is as follows [22]:

(8) $$\begin{array}{c} \Psi_{a,b}\left(t\right)={\left|a\right|}^{-1/2}\Psi \left(\frac{t-b}{a}\right). \end{array}$$

The reason for the wavelet transform is to represent an arbitrary function *f* as a superposition of a small wave. At the same time, the original function can be reconstructed from the wavelet decomposition. Under constraints, Ψ has “sufficient” decay properties, namely,

(9) $$\begin{array}{c} \int \frac{{\left|\Psi \left(\omega \right)\right|}^{2}}{\left|\omega \right|}d\omega \;<\infty , \end{array}$$

where Ψ(*ω*) is the Fourier transform of *ψ*(*t*); it can decompose and refactor arbitrary functions. Typically, the wavelet transform uses a discrete form. Considering that we are primarily interested in a signal with a continuous resolution of 2, it can define discrete wavelet transform by *a*=2^*m*^ and *n*2^*m*^, where *m* and *n* are integers [23]. Then, the decomposition formula of the wavelet becomes the following form:

(10) $$\begin{array}{c} f\left(t\right)=\sum_{m,n}c_{m,n}\psi_{m,n}\left(t\right). \end{array}$$

Among them, *ψ*_*m*,*n*_(*t*)=2^−*m*/2^*ψ*(2^−*m*^*t* − *n*), *ψ* has very special forms, making *ψ*_*m*,*n*_(*t*) form a standard orthonormal basis, namely,

(11) $$\begin{array}{c} c_{m,n}=\langle f,\psi_{m,n}\rangle =\int \psi_{m,n}\left(t\right)f\left(t\right)dt. \end{array}$$

In order to do this, we need to construct a scaling function *φ*, as well as its dilation and transformation form *φ*_*m*,*n*_(*t*)=2^−*m*/2^*φ*(2^−*m*^*t* − *n*). Let *h*_*n*_=2^1/2^∫*φ*(*t* − *n*)*φ*(2*t*)*dt*, *g*_*l*_=(−1)^*l*^*h*_1−*l*_. It can be seen that the following recurrence relation exists between the coefficients *c*_*m*,*n*_ and *a*_*m*,*n*_:

(12) $$\begin{array}{c} \begin{array}{c} c_{m,n}=\sum_{k}g_{2n-k}a_{m-1,k},\\ a_{m,n}=\sum_{k}h_{2n-k}a_{m-1,k}. \end{array} \end{array}$$

If function *f* is given in sampled form, these sampling processes are then interpreted as the highest order resolution approximation coefficients *a*_0,*n*_. A rough approximation of the function can be recursively computed by combining down sampling and filtering operations. Due to their relation to the orthogonal wavelet basis, these filters can provide perfect reconstructions such as follows:

(13) $$\begin{array}{c} a_{m-1,l}\left(f\right)=\sum_{n}\left[h_{2n-1}a_{m,n}\left(f\right)+g_{2n-1}c_{m,n}\left(f\right)\right]. \end{array}$$

FIR filters of finite length have been constructed as wavelet transforms, and the same filters are used for reconstruction and decomposition. However, these filters are not symmetric, so the relationship between them is nonlinear [24]. In order to ensure a perfect refactoring, the following calculations are required:

(14) $$\begin{array}{c} {\tilde{g}}_{n}={\left(-1\right)}^{n}h_{1-n},\\ g_{n}={\left(-1\right)}^{n}h_{1-n},\\ \sum_{n}h_{n}{\tilde{h}}_{n+2k}=\delta_{k,0}. \end{array}$$

The relationship between the filter and the wavelet function and the scale function is expressed in the following formula:

(15) $$\begin{array}{c} \varphi \left(t\right)=\sum_{n}h_{n}\varphi \left(2t-n\right),\\ \tilde{\varphi }\left(t\right)=\sum_{n}{\tilde{h}}_{n}\tilde{\varphi }\left(2t-n\right),\\ \psi \left(t\right)=\sum_{n}g_{n}\varphi \left(2t-n\right),\\ \bar{\psi }\left(t\right)=\sum_{n}{\tilde{g}}_{n}\overset{⟶}{\varphi }\left(2t-n\right). \end{array}$$

#### 3.1.5. Image Fusion with Multiple Sources Based on MGA Method

The Multivariate Geome Analysis (MGA) theory provides better feature extraction functionality in feature representation than traditional pyramidal transformations and small wave transitions. This has led to a breakthrough in multisource image integration. Efficient, multidimensional representations of images are used in image processing such as compression, noise, and image consolidation. Contourlet Transform (CT) is one of the latest technologies with multiple sizes. In addition to the actual 2D image enhancement filter, the flexible directional filter also captures the inherent geometry of the image and provides smooth image shifting in NSCT. The Gibbs phenomenon caused by coefficient correction has been greatly improved over CT as filter interpolation replaces image extraction.

### 3.2. Framework Based on Grayscale Image Fusion

To simplify and clarify this process, a pair of CT and MR brain images is shown. The specific integration process is as follows:

 
*Step 1*. The medical images are resolved to be merged using the NSST algorithm. aA and bB are low-pass subband coefficients. *cA*^*s*,*j*^ and *cB*^*s*,*j*^ are the high-pass subband coefficients at *l* in layer *s*.
 
*Step 2*. Low-cut parts are processed using activity level measurements based on energy storage and detail capture.
 
*Step 3.* The high-pass part is integrated using the activity level metric based on IQPSO-PCNN.
 
*Step 4*. The inverse NSST algorithm is used to generate the final fusion result from the fused low-pass and high-pass parts.

Consisting of a geometric and multidimensional imitation system, it offers advantages such as anisotropy, relatively simple structures, efficient data processing, and no directional orientation and is often used as a multidimensional decomposition tool for images [25]. When the dimension *n* = 2, an imitation system with compound extension can be represented as follows:

(16) $$\begin{array}{c} \psi_{AB}\left(\psi \right)=\left\{\psi_{j,k,l}\left(x\right)=\left|\left(B^{l}A^{j}x-k\right)\right|,j,l\in Z,k\in Z^{2}\right\}. \end{array}$$

Among them, *ψ* ∈ *L*^2^(*R*^2^), *L* is the space that can be integrated, matrices *A* and *B* are inverse matrix of the second class, and |det*B*|=1. An arbitrary *f* ∈ *L*^2^(*R*^2^) is satisfied if *ψ*_*AB*_(*ψ*) is a Parseval frame. The specific form is as follows:

(17) $$\begin{array}{c} {\sum_{j,k,l}\left|\langle f,\psi_{j,k,l}\rangle \right|}^{2}={\left\|f\right\|}^{2}. \end{array}$$

Among them, *ψ*_*AB*_(*ψ*) is the set of basis functions, A represents the anisotropic matrix of multidimensional intervals, *s* is the shear matrix used for direction analysis, and *j*, *l*, and *k* are the parameter values for scale, orientation, and translation, respectively. The representation of matrices *A* and *B* is as follows:

(18) $$\begin{array}{c} A=\left[\begin{array}{cc} a & 0\\ 0 & \sqrt{a} \end{array}\right],B=\left[\begin{array}{cc} 1 & s\\ 0 & 1 \end{array}\right]. \end{array}$$

Among them, when *a* = 4 and *s* = 1, the synthesized wavelet is a shear wave.

(19) $$\begin{array}{c} {\hat{\psi }}_{j,l,k}^{0}\subset \left\{\xi_{1},\xi_{1}|\xi_{1}\in \left[-2^{2j-1},-2^{2j-4}\right]\cup \left[-2^{2j-4},-2^{2j-1}\right],\left|\frac{\xi_{2}}{\xi_{1}}+l2^{-j}\right|\leq 2^{-j}\right\}. \end{array}$$

For different decomposition scales, each element ${\hat{\psi }}_{j,l,k}^{0}$ is supported on a pair of trapezoidal regions, its size is about 2^2*j*^ × 2^*j*^, and the slope in the line direction is *l*2^−*j*^ [26].

## 4. Results and Analysis

### 4.1. Analysis of Multisource Image Fusion Technology

#### 4.1.1. Objective Evaluation Indicators

There is no unified evaluation standard for quality evaluation based on fused images. In most cases, people evaluate image quality based on subjective impressions; this can lead to unconvincing assessment results. As a result, the obtained image is validated from four different angles, and the fusion effect is evaluated globally, including the quality index of local importance (*Q*_*o*_), Piella structural similarity measure (*Q*_*w*_), entropy (EN), and standard deviation (SD).

#### 4.1.2. Parameter Setting

Set the NSST explosion level to 4. In the IQPSO algorithm, set the number of dimensions of the particles to 5 (*D* = 5), the total number of particles to 20 (*N* = 20), and the number of iterations to 50(Maxtime = 50). In the PCNN algorithm, the number of iterations is fixed at 100, link matrix W_ijkl_ = [0.7071,1,0.7071;1,O, 1;0.7071,1,0.7071].

### 4.2. Analysis of Grayscale Image Fusion Results

This section uses the grayscale image fusion framework to test grayscale image fusion in four different modes, including MR-TI and CT, MR-PD and MR-T1 grayscale images, MR-T1 and MR-T2, as well as MR-T2 and MR-Gad, and provides qualitative analysis and quantitative description of the algorithm.

#### 4.2.1. MR-T1 and CT Experimental Results Analysis

**Figure 4:**
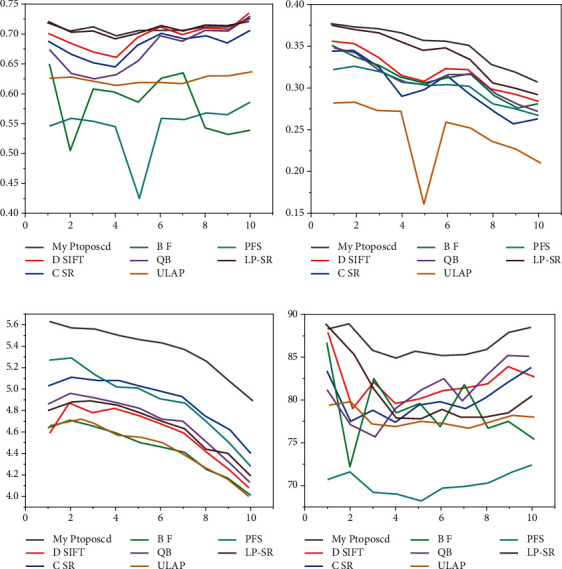
Line chart of MR-T1 and CT evaluation indicators.

Figure 4 shows the results of the experiments for the MR-T1 and CT datasets. While the LP-SR and ULAP methods give better results than other methods, the extraction of detail and conservation of energy are not as good as described in this chapter. While the mean is not optimal for some images, it is better than others, indicating that the algorithm has the best visual results and objective ratings [27].

Figure 4(a) is the *Q*_*w*_ result, Figure 4(b) is the *Qo* result, Figure 4(c) is the EN result, and Figure 4(d) is the SD result.

#### 4.2.2. Analysis of MR-T1 and MR-PD Experimental Results

**Figure 5:**
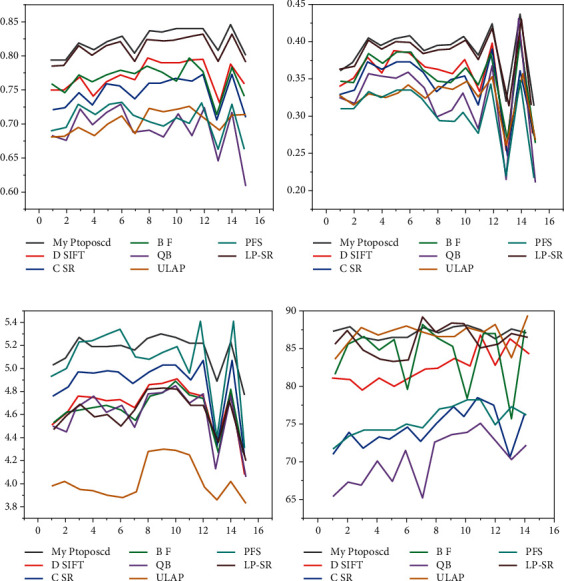
The line chart of the evaluation indicators of MR-T1 and MR-PD.

Figure 5 shows the laboratory analysis for MR-T1 and MR-PD. The BF and DSIFT methods have a significant resolution limitation such that an adjusted image cannot store distinct information from the source image. LP-SR and ULAP algorithms work well for soft tissues of the brain, but there is still a little less contrast in some areas. It can be clearly observed in the figure that the author's method slightly outperforms other algorithms on EN, *Q*_*w*_, and *Q*_*o*_ metrics, indicating that the algorithm is better than others in terms of structural similarity, energy storage, and detail extraction [28].

Figure 5(a) is the *Q*_*w*_ result, Figure 5(b) is the *Q*_*o*_ result, Figure 5(c) is the EN result, and Figure 5(d) is the SD result.

## 5. Conclusion

Multisource image consolidation technology combines images captured by different types of sensors or images of the same type of sensor with different parameters, reducing the redundancy and randomness of the images and improving the image quality and the characteristic information of the image. Multisource image fusion technology is an important branch of computer vision and a research hot spot in the field of image understanding, and it is widely used in the fields of remote sensing, medicine, and science. From this, it can be seen that detailed and in-depth research on multisource image fusion technology is carried out, and it has profound and important significance. Disclosed is a medical image fusion algorithm based on edge conservation and scatter representation enhancement, based on the idea of edge information and scatter representation, which first uses the NSST algorithm to decompose the image at several scales and obtain low frequency and high-frequency subbands. For low frequency subbands which are often affected by detailed information by the scatter representation based fusion method, this paper proposes an improved scatter representation method for low frequency subband fusion. The algorithm effectively improves the ability of the algorithm to conserve energy by eliminating detailed training set features in learning the dictionary through the Sobel operator and guide filter. Since the high-frequency subbands of different medical modalities have different edge information characteristics at the boundary, the author proposes a PCNN fusion strategy based on edge preservation; this strategy can well deal with the problem of blockiness in the fusion boundary. The experimental results show that this method can fuse grayscale images; when the grayscale image and the color image are fused, the objective evaluation index and the subjective visual effect are better than the comparison algorithm.

## Data Availability

The statistical data used to support the findings of this study are available from the corresponding author upon request.
